# Trends in targeted prostate brachytherapy: from multiparametric MRI to nanomolecular radiosensitizers

**DOI:** 10.1186/s12645-016-0018-5

**Published:** 2016-07-04

**Authors:** Alexandru Mihai Nicolae, Niranjan Venugopal, Ananth Ravi

**Affiliations:** 1 Odette Cancer Centre, Sunnybrook Health Sciences Centre, 2075 Bayview Ave, Toronto, ON M4N3M5 Canada; 2 Saskatoon Cancer Centre, 20 Campus Drive, Saskatoon, SK S7N4H4 Canada

**Keywords:** Prostate cancer, Brachytherapy, Dose escalation, Hypofractionation, Multiparametric MRI, Gold nanoparticles, Dominant intraprostatic lesion

## Abstract

The treatment of localized prostate cancer is expected to become a significant problem in the next decade as an increasingly aging population becomes prone to developing the disease. Recent research into the biological nature of prostate cancer has shown that large localized doses of radiation to the cancer offer excellent long-term disease control. Brachytherapy, a form of localized radiation therapy, has been shown to be one of the most effective methods for delivering high radiation doses to the cancer; however, recent evidence suggests that increasing the localized radiation dose without bound may cause unacceptable increases in long-term side effects. This review focuses on methods that have been proposed, or are already in clinical use, to safely escalate the dose of radiation within the prostate. The advent of multiparametric magnetic resonance imaging (mpMRI) to better identify and localize intraprostatic tumors, and nanomolecular radiosensitizers such as gold nanoparticles (GNPs), may be used synergistically to increase doses to cancerous tissue without the requisite hazard of increased side effects.

## Background

Recent studies of the radiobiological properties of prostate cancer cells demonstrate a low alpha/beta ratio; this suggests that hypofractionation—the delivery of larger radiation doses in a smaller number of treatment cycles—may offer the best chance of long-term disease control for localized prostate cancer (Brenner and Hall 1999; Brenner et al. 1998; Vogelius and Bentzen 2013; Carbrera and Lee 2013; Sanfilippo and Cooper 2014). Brachytherapy (BT), the temporary or permanent implantation of small, gamma-emitting radioactive sources directly within cancerous tissue, is a highly effective method for delivering extremely hypofractionated radiation to the prostate (Sanfilippo and Cooper 2014; Yoshioka et al. 2011; Ritter et al. 2011; Tselis et al. 2013). The proximity of the radioactive sources to the cancerous lesions, as well as their placement interstitially within the prostate gland, ensures that the radioactive sources move with the organ, thereby limiting the impact of organ motion on the accuracy of the treatment compared to external beam radiation therapy (EBRT). This phenomenon, along with the rapid dose fall-off beyond the edge of the prostate, enables improved, localized dose escalation (Lee 2009). Several clinical studies demonstrate excellent long-term biochemical disease control rates across risk groups, for both permanent and temporary brachytherapy, and either alone or in combination with EBRT or androgen deprivation therapy (ADT) (Tselis et al. 2013; Ishiyama et al. 2014; Martinez et al. 2010; Morris et al. 2015a, b; Hoskin 2012). A biochemical control and metastasis-free survival rate at 5 years of 94 and 98 %, respectively, was shown by Tselis et al. 2013who evaluated 351 localized prostate cancer patients treated with high-dose-rate (HDR) brachytherapy (Tselis et al. 2013). Preliminary results demonstrated by the large multi-institutional ASCENDE-RT trial show similar long-term control for BT as a boost treatment with EBRT (Morris et al. 2015a, b). In addition to long-term survival benefits, BT also offers significant quality-adjusted life-years (QALYs) benefits over both EBRT alone and radical prostatectomy (RP), the surgical removal of the prostate (Hayes 2010; Steuten and Retel 2013). As a result of the significant benefits of delivering dose-escalated brachytherapy, it has been routinely recommended, either alone or in combination with other treatment modalities, for the treatment of organ-confined disease by the American Brachytherapy Society (ABS), as well as the Groupe Européen de Curithérapie (GEC), and the European Society for Radiotherapy and Oncology (ESTRO) (Davis et al. 2012; Yamada et al. 2012; Hoskin et al. 2013).

Despite the many advantages BT offers, there is growing evidence that increasing radiation doses, without improving dose conformity or targeting accuracy, results in unacceptable patient toxicities (Tselis et al. 2013; Morris et al. 2015a, b; Helou et al. 2014). It has been hypothesized that improving the sensitivity and specificity with which radiation is targeted to prostatic lesions through novel imaging and therapeutic modalities may remove this upper limit on allowable dose escalation (Helou et al. 2014). In this regard, the rapidly expanding fields of multiparametric magnetic resonance imaging (mpMRI) and targeted gold nanoparticles (GNPs) are two of the latest methods that hold the most promise for enabling safe dose escalation (Wallace et al. 2013; Ghai and Haider 2015).

Multiparametric MRI has recently emerged as the imaging modality of choice for detection of localized prostate cancer (supplementing the histopathological information provided by ultrasound-guided biopsies) and has become part of the standard of care for the diagnosis, localization, and staging of prostate cancer, largely due to its superior soft tissue contrast and supplementary functional information (Ghai and Haider 2015; Lawrentscuk and Fleshner 2009; Panebianco et al. 2015; Scheenen et al. 2015; Weinreb et al. 2016). The addition of multiple functional imaging sequences to the anatomical information provided by T2-weighted (T2W) static MR images—including diffusion-weighted imaging (DWI), dynamic contrast-enhanced imaging (DCEI), and magnetic resonance spectroscopic imaging (MRSI)—produce a wealth of additional information to aid in the delineation of active disease (Panebianco et al. 2015; Maneti et al. 2014; Verma et al. 2012; DiBiase et al. 2002). More recently, mpMRI has garnered attention in prostate brachytherapy treatment planning, for improving both prostate gland and intraprostatic lesion localization (Gomez-Iturriaga et al. 2016; Marks et al. 2013; Kaplan et al. 2010; Wu et al. 2014; Menard et al. 2004). The advantages provided by co-registering mpMRI with intra-operative transrectal ultrasound (TRUS) within the prostate BT workflow could significantly reduce the current limitations imposed on dose escalation; this review will examine mpMRI with relation to both its diagnostic and target localization potential for BT (Gomez-Iturriaga et al. 2016). Additionally, the development of intra-operative MRI-only workflows for BT will be examined.

The enhancement of the therapeutic ratio for prostate brachytherapy may also be achieved through the use of radiosensitizers to selectively improve radiation dose delivery to cancerous tissue. Targeted gold nanoparticles (GNPs), due to their physical, radiation, and pharmacokinetic properties, are well suited to BT applications with the potential to boost local radiation doses to levels unimaginable with EBRT or conventional BT alone (Ngawa et al. 2013; Lechtman et al. 2013; Arnida and Ghandehari 2010; Babaei and Ganjalikhani 2014). The addition of radiosensitizers to the BT workflow, in combination with the high spatial resolution of MRI, could significantly improve dose escalation while further sparing patients the associated increase in toxicities. The currently available clinical studies and future potential of these novel nanomolecular agents will be examined in a BT framework.

By synergistically combining the improved image guidance of mpMRI, and the selective targeting provided by nanomolecular radiosensitizers, new treatment paradigms within the BT workspace can also be realized. Single-treatment BT with biological doses beyond what is currently achievable, a greater focus on targeting and treatment of intraprostatic lesions or single dominant intraprostatic lesions (DILs), and improvements in focal and salvage therapy are all potential new areas that may see a rapid improvement with adoption of new dose-escalation methods. Many of these novel directions within the BT space will be covered in this review.

The overall goal of this review is to evaluate the current landscape of dose-escalated prostate brachytherapy in its present form and examine ways in which mpMRI guidance and nanoparticle radiosensitization can selectively improve dose escalation in the future. The constantly changing research and clinical landscape of targeted therapy for prostate cancer makes it difficult to perform an exhaustive review on this rapidly evolving field; this review represents only an instance of the literature at the time of publication.

## Clinical overview of prostate brachytherapy

The two most common methodologies for delivering hypofractionated radiation for localized prostate cancer are low-dose-rate (LDR) and high-dose-rate (HDR) BT. LDR, or permanent implant BT, involves the permanent placement of 80–100 radioactive substances within the interstitial space of the prostate gland. High-dose-rate (HDR) BT, by comparison, involves the temporary implantation of hollow catheters into the prostate through which a highly radioactive source is remotely passed for a short period of time; the transit time of the source within the catheters determines the magnitude of the delivered dose. Both workflows generally follow a similar planning framework as recommended by both ABS and GEC-ESTRO guidelines (Davis et al. 2012; Yamada et al. 2012; Hoskin et al. 2013). The components of a typical BT patient pathway are as follows: (1) diagnosis of localized prostate cancer, (2) pre-operative or intra-operative treatment planning and treatment delivery, (3) post-implant quality assurance.

### Diagnosis using MRI

Traditionally, definitive diagnosis of localized prostate cancer relied on the TRUS-guided sextant biopsy for identification of cancerous tissue; this approach alone was found to leave up to 15 % of cancers undetected and was prone to sampling error (Norberg et al. 1997). The information from TRUS biopsy is now routinely correlated with imaging information provided by multiparametric MRI (mpMRI); this offers a clearer representation of disease foci and extraprostatic disease extension as well as improved biopsy sampling and characterization as part of staging (Lawrentscuk and Fleshner 2009; Kaplan et al. 2002; Cirillo et al. 2008; Pullini et al. 2016). In 2012, the *prostate imaging*–*reporting and data system* (PI-RADS v.1, updated in 2015/2016) was developed to standardize the approach to diagnosis and reporting of mpMRI for prostate cancer (Weinreb et al. 2016); several recommendations were made. Diagnosis of prostate cancer should be performed using T2-weighted fast spin echo (T2W-FSE) sequences on a 1.5–3T MRI scanner, the latter being preferred due to its higher signal-to-noise ratio, along with inclusion of functional DCEI, DWI, and potentially MRSI sequences. A high degree of visibility of the prostate peripheral zones, along with urethral and rectal structures, seminal vesicles, and prostatic capsule (extra- and intracapsular disease) is typically required. Each imaging sequence of the mpMRI acquisition contributes supplementary information that facilitates diagnosis and/or staging of localized disease; the PI-RADS reporting system further standardizes this approach according to the respective imaging sequence.

**Fig. 1:**
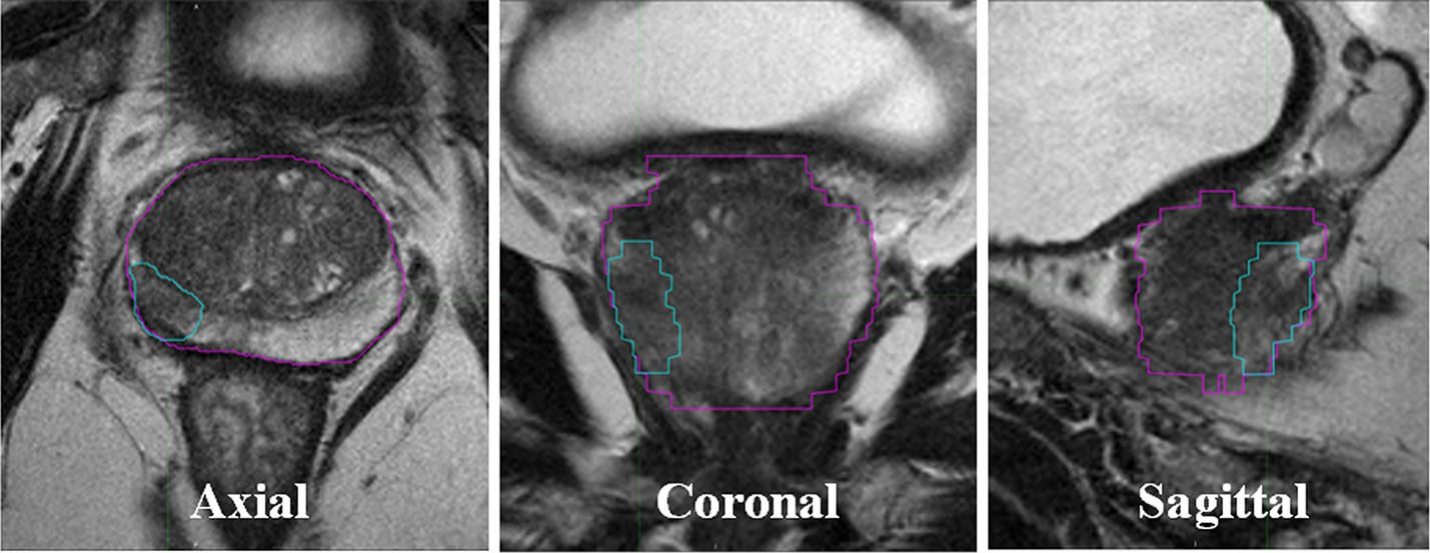
Multiplanar T2-weighted fast-spin echo (T2W-FSE) images (axial, coronal, and sagittal midplanes) of a patient with localized prostate cancer treated with EBRT (45 Gy/25), followed by an HDR BT boost (15 Gy/1). The prostate (*purple*) and dominant intraprostatic lesion (*light blue*) are not readily visible without supplementary functional imaging

T2W-FSE images (see Table 1 for recommended PI-RADS parameters) are typically used to discriminate between zonal anatomies of the prostate and discern normal tissue from various abnormalities; however, a number of benign conditions may mimic the T2W appearance of prostate cancer including benign prostatic hyperplasia (BPH) or prostatitis (Kitajima et al. 2010). Intraprostatic cancerous tissue conversely may also be difficult to detect on this imaging alone; Fig. 1 shows a typical example of a multiplanar T2W-FSE image of the prostate and surrounding anatomy. The limitations of purely anatomic imaging necessitate the supplementary use of functional imaging sequences.

**Table 1:** PI-RADS recommendations for mpMRI sequence parameters

Imaging sequence	Slice (mm)	FOV (cm)	In-plane dimensions (mm)	TR/TE (msec)	Additional	Contrast
T2W	3	12–20	≤0.7 × ≤0.4	Not specified	–	–
DWI	≤4	16–22	≤2.4 × 2.5	≤90/ ≥3000	*b*_min_ = 50–100 s/mm^2^ *b*_max_ = 800–1000 s/mm^2^	–
DCEI	3	Entire prostate + SV	≤2 × 2	<100/ <5	Observation time ≥2 min	0.1 mmol/kg GBCA; injection rate = 2–3 cc/sec

Since MRSI is still investigational, PI-RADS does not specify MRSI as standard for mpMRI sequences in prostate cancer diagnosis

**Fig. 2:**
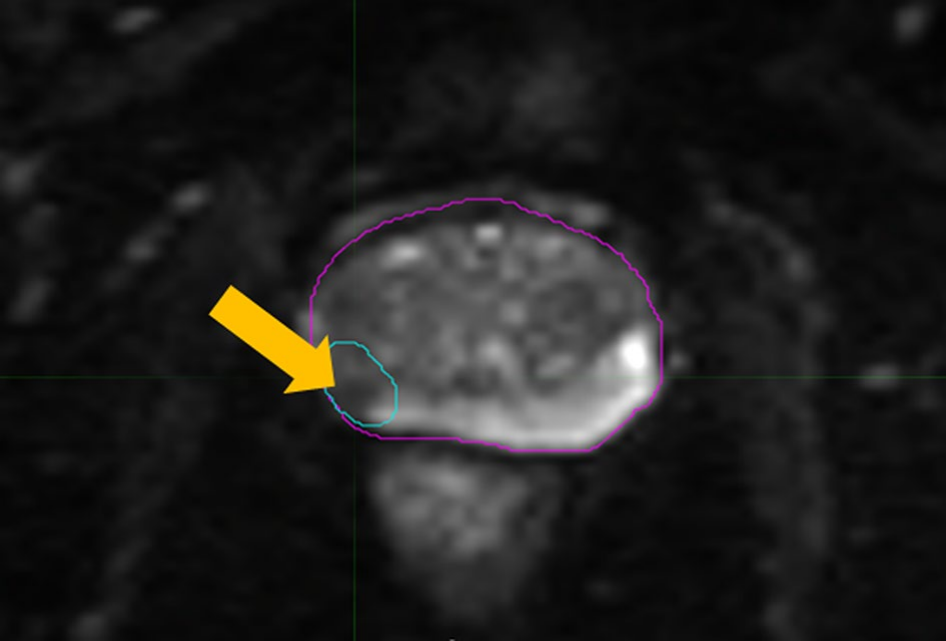
Diffusion-weighted imaging (DWI) of an axial midgland plane with high *b* value ADC map. Areas of higher water diffusion rates appear brighter on T2 imaging. Significant cancers may present with restricted diffusion rates and are seen as areas of hypointense signal on the ADC map (*arrow*). The focal lesion is contoured for clarity

DWI, a functional method of measuring random water molecule diffusion rates within tissue, is typically used to supplement T2W-FSE acquisitions. Prostate cancers present with restricted diffusion rates relative to the surrounding normal prostatic tissue, a phenomenon that is represented through apparent diffusion coefficient (ADC) maps computed at each image voxel (Weinreb et al. 2016; Kim et al. 2010). *B* values—an indicator of the rate of diffusion that is captured by the image dataset—have recommended ranges between 0 and 1000 s/mm^2^; larger *b* values are used to detect slower diffusion rates of water molecules (Kim et al. 2010). The inclusion of DWI sequences with T2W imaging improves detection of prostate cancer, particularly in the peripheral zone tumors (Haider et al. 2007). Figure 2 shows a typical DWI axial sequence of the prostate.

**Fig. 3:**
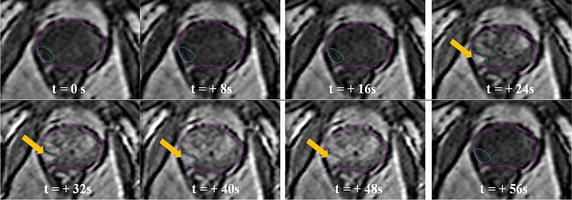
Axial midgland sequence showing dynamic contrast-enhanced imaging (DCEI) acquired using T1W-FSE sequences over a 1 min period. Gadolinium-based contrast agent (GBCA) is administered intravenously at an injection rate of 2–3 cc per second; lesion enhancement may appear as early as 10 s following injection. Enhancement of the DIL is shown in frames 4 through 7 (*arrows*), followed by a washout phase where the signal dissipates

DCEI obtained by acquiring T1W image sequences during administration of an intravenous gadolinium-based contrast agent (GBCA), is used to further supplement the information obtained from both T2W and DWI sequences. DCEI takes advantage of cancer angiogenesis, a process that increases vascular density and permeability within tumors, to visualize prostatic regions of increased uptake of the GBCA (Verma et al. 2012; Singanamalli et al. 2016). Figure 3 shows the differential uptake of GBCA within areas of active tumor (the DIL), over a set of sequential axial frames. The addition of DCEI to the mpMRI sequence has demonstrated improvements in sensitivity and lesion detection accuracy (Kitajima et al. 2010; Alonzo et al. 2016).

**Fig. 4:**
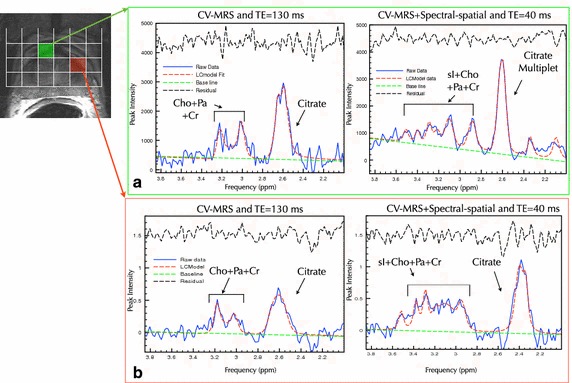
Magnetic resonance spectroscopic imaging (MRSI) spectra obtained using a conformal voxel approach for areas of normal tissue (*green voxel*), and suspected abnormality (*red voxel*) and for short (TE = 40 ms), and long (TE = 130 ms) echo times. A spectral-spatial RF pulse sequence was used. In **a** normal voxels are displayed demonstrating the appearance of short TE metabolites (i.e., sI—scyllo-inositol) not evident at longer echo times. Similarly, in **b** there is a decrease in relative concentration of citrate to choline over voxels with suspected cancer

Magnetic resonance spectroscopic imaging (MRSI), while not as widely adopted as DWI or DCEI, is gaining interest as a valuable technique for non-invasively determining the biochemical concentrations of biomarkers associated with prostate cancer (Kurhanewicz et al. 2002; Picket et al. 2004; Pouliot et al. 2004). MRSI may be more specific in differentiating benign conditions from actively metabolic prostate cancer and may provide metabolic information associated with tumor progression (Kobus et al. 2014). In vivo prostate MRSI utilizes the relative or absolute concentrations of the metabolites such as choline, polyamines, creatine and citrate, as cancer specific biomarkers, with 98 % of cancers demonstrating an elevated (choline + creatine)/citrate ratio greater than the ratio for normal tissue (Fig. 4) (Westphalen et al. 2008; Scheenen et al. 2015). Addition of the functional information provided by MRSI could potentially improve diagnosis, staging, and disease localization for BT. The diagnostic value of combined magnetic resonance imaging and spectroscopic techniques has encouraged radiologists and oncologists to include it increasingly for clinical use.

### Pre- and intra-operative image guidance and treatment planning

Once diagnosis and staging of prostate cancer have been completed (and BT is selected as a treatment option), the identification of critical target and normal tissue structures is made. During this phase, the target, typically the entire prostate gland and/or focal lesions, and organs at risk (OARs) are identified for treatment planning (Thomadsen et al. 2012; Yu et al. 1999). Historically, CT, TRUS, and more recently mpMRI, have been used to identify critical structures (Nath et al. 2009; Metcalfe et al. 2013; Rischke et al. 2013). Clinical use of CT or TRUS has traditionally been the mainstay of pre-treatment imaging for target identification, but recent improvements in the availability of diagnostic MRI scanners have improved access significantly to mpMRI for this purpose (Davis et al. 2012; Yamada et al. 2012; Nag et al. 2000; Mayer et al. 2016). Several studies have pointed to the subjectivity of contouring on conventional imaging modalities. Better imaging methods are being heralded as a means to provide objectivity to this aspect of the planning process (Rischke et al. 2013; Steenbergen et al. 2015; Fiorino et al. 1998).

#### Pre-operative guidance prior to MRI

**Fig. 5:**
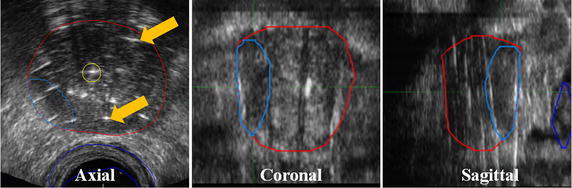
TRUS axial, coronal, and sagittal midgland planes for a patient treated with TRUS-guided HDR BT. The live TRUS images were co-registered with the contours obtained from mpMRI to yield the prostate (*red*) and DIL (*light blue*) contours. The difficulty in identifying distal catheters compared to proximal catheters is also apparent (*arrows*)

CT-guided BT planning represents one of the earliest attempts to use volumetric imaging to accurately identify targets and OARs, as well as plan radioactive source positions, with good outcomes; Koutrouvelis et al. (2000) reported prostate-specific antigen (PSA) <2 ng/mL in 90 % of patients (*n* = 301) at median 26 month follow-up after being treated with permanent implant CT-guided BT (Koutrouvelis et al. 2000). Intra-operative TRUS-guided BT is rapidly growing in adoption largely due to its lower cost, widespread availability, and real-time guidance, and has allowed implant guidance during the BT procedure in addition to target localization. Stone et al. (2007), pioneers of the TRUS-guided permanent implant approach, reported excellent long-term toxicity outcomes of 325 patients (Stone and Stock 2007; Crook et al. 2011). TRUS-guided BT is now the recommended standard of care for prostate BT by both ABS and GEC-ESTRO guidelines for both LDR and HDR implants (Davis et al. 2012; Yamada et al. 2012; Hoskin et al. 2013). Despite these many advantages, significant TRUS artifact still make identification of the target and OARs highly subjective (Fig. 5) (Xue et al. 2006; Solhjem 2004). Further identification of DILs for dose escalation is simply not feasible using TRUS guidance alone, and a workflow incorporating mpMRI fusion with TRUS imaging is commonly required.

#### Pre-operative MRI for treatment planning

MRI, in addition to its diagnostic capability, has been recognized as the ideal imaging modality for soft tissue prostate delineation, as well as for discrimination of nearby normal tissues (Dinh et al. 2016). Using the information obtained from mpMRI radiation, oncologists can identify the prostate, focal lesions, and nearby healthy tissues (urethra and rectum, commonly) with greater confidence than using TRUS or CT imaging.

However, since the information from mpMRI is typically only available in a pre-operative setting, an additional imaging modality must be co-registered to obtain contours of the target and OARs during the planning stage. Following an initial pre-treatment mpMRI the dataset is co-registered with live intra-operative TRUS, a technique adapted from advances in TRUS-guided biopsies (Kaplan et al. 2002; Marks et al. 2013). Delineated contours from the mpMRI are then propagated onto the live TRUS images; this approach has allowed improved targeting of the prostate, and even potentially DILs, without altering the intra-operative imaging modality (DiBiase et al. 2002; Marks et al. 2013; Crook et al. 2014). Unilateral focal disease has been treated using DCE imaging to contour the DIL with the intent of focal dose escalation. Images from the mpMRI were transposed onto the intra-operative TRUS. An average of 20–30 % dose escalation to the DIL was feasible using this approach (Crook et al. 2014). More recently, Gomez-Iturriaga et al. (2016) were able to treat 15 patients with a single MR-TRUS-guided HDR boost of 15 Gy; the DIL was treated to 18.75 Gy. At a median follow-up of 18 months, none of the patients developed grade ≥3 urinary toxicity (Gomez-Iturriaga et al. 2016). Although registration errors may occur due to the imperfect correlation of MRI to live TRUS imaging, many commercially available deformable registration algorithms have been developed to improve this process and are a growing area of interest (Sparks et al. 2013). Target and OAR identification continue to pose a challenge for mpMRI-fused BT workflows; as a result, the use of intra-operative MRI guidance for BT has garnered significant interest in an attempt to alleviate this problem.

#### Intra-operative guidance prior to MRI

**Fig. 6:**
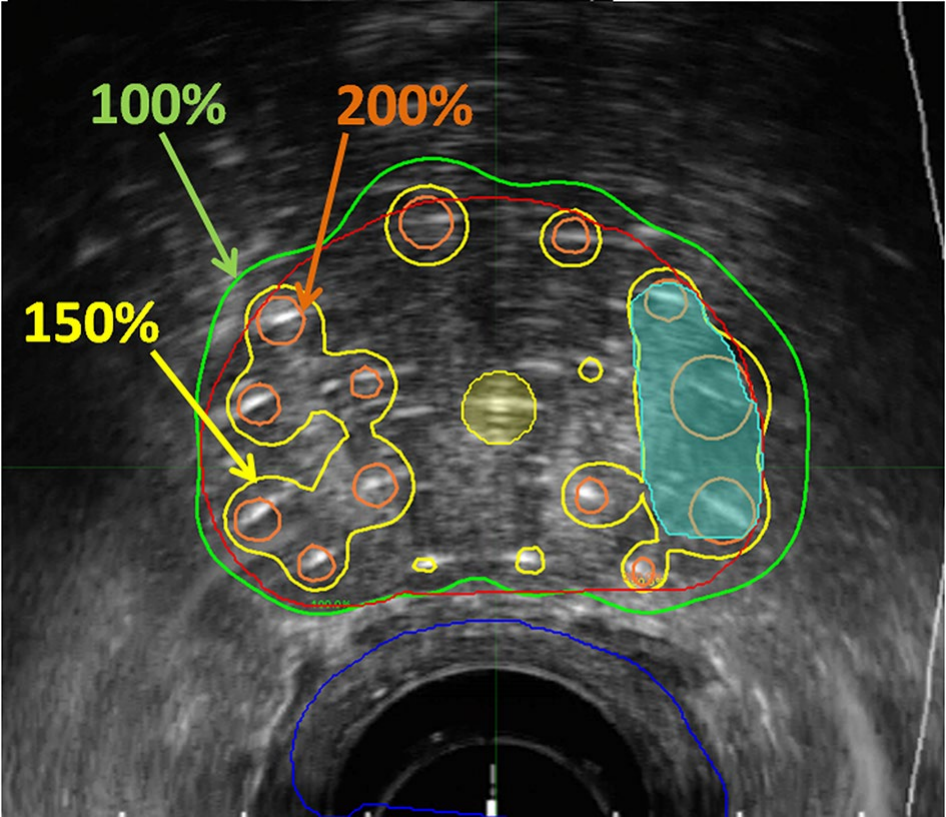
Typical HDR prostate BT treatment plan showing axial midgland plane for a patient treated with 19 Gy. Twelve catheters (hyperechoic regions) were implanted and dwell times optimized using Oncentra Prostate (Elekta AB, Stockholm, Sweden). The organ contours are the prostate (*red*), urethra (*light blue*), focal lesion (*light teal*), and rectum (*dark brown*). The isodose lines are represented as a percentage of the prescription dose (*arrows*)

Current GEC-ESTRO and ABS guidelines for prostate brachytherapy recommend intra-operative TRUS imaging for visualization of the prostatic capsule, nearby anatomy, and implant guidance. Interpretation of TRUS images is highly subjective, leading to difficulties in target, OAR, and source identification during implantation. Figure 5 outlines some of the difficulties in identification of catheter trajectories for an HDR prostate BT procedure. For HDR BT, once catheter identification has been completed, an optimization algorithm is used to determine the optimal dwell times of the radioactive source within each catheter with the goal of maximizing the radiation dose to the target(s) while minimizing the dose to normal tissues (Fig. 6). A similar process in LDR BT is used to determine the configuration of implanted sources within the prostate gland.

#### Intra-operative guidance using MRI

The use of intra-operative MRI guidance for prostate brachytherapy arose from earlier iterations with interventional TRUS biopsies performed in both open and closed-bore MRI scanners (D’Amico et al. 2000; Tokuda et al. 2012). When applied to both LDR and HDR brachytherapy, this approach enabled the monitoring of implanted sources and/or needles with respect to the soft tissue boundaries of the prostate and normal tissues. These workflows typically mimic current intra-operative BT workflows using TRUS guidance. Prostate or DIL identification is done by a radiologist in the MRI interventional suite with the patient anesthetized in the scanner bore. Intra-operative approaches to MRI-guided BT involving low-field (0.2–0.5 T) (Cormack et al. 2000; Ares et al. 2009) and high-field closed-bore (1.5–3 T) (Menard et al. 2004; Susil et al. 2004) have been evaluated clinically with encouraging early results. Low-field, open-bore systems offer improved interventional access to the patient, but at the cost of decreased imaging quality due to the lower field strength; additionally, the low field strength significantly limits application of DCE imaging and other functional techniques. Higher field strength, closed-bore magnets, although creating challenges for interventional approaches due to the limited access to the patient, offers superior image quality and are more readily available in clinical settings (as 1.5 or 3 T units).

The intra-operative workflow outlined by Menard et al. (2004) is an excellent example of an MRI-dedicated HDR workflow using a closed-bore, high-field strength (1.5 T) MRI (Menard et al. 2004). Patients were placed in the left lateral decubitus position into the scanner bore and anesthetized using general anesthesia. An endorectal coil was inserted to improve visualization of the pelvic anatomy and scout MRI images were obtained to localize the treatment site. Catheters (with metallic, MR-safe obturators) were implanted by removing the scanner table from the scanner isocenter, inserting the catheter, advancing the table back to the isocenter, and then re-acquiring FSE sequences to evaluate the catheter positions (on both axial and sagittal planes). Early clinical results have been promising; however, the limitations of the closed-bore procedure increase the procedure time significantly over the current standard TRUS-guided techniques (Menard et al. 2004; Ares et al. 2009).

### Post-implant quality assurance

**Fig. 7:**
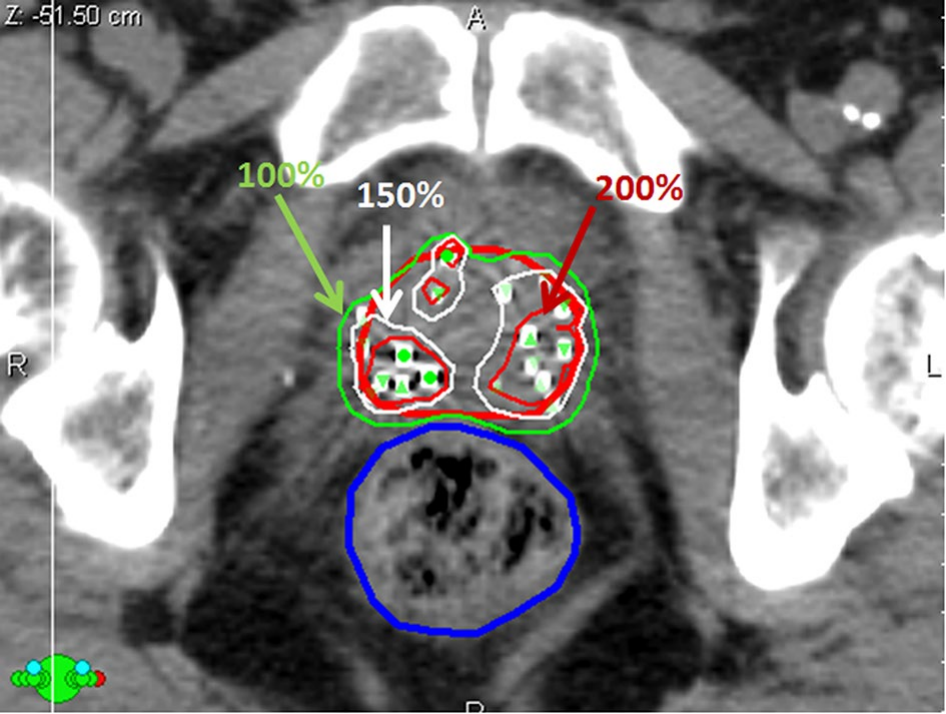
Post-implant dosimetric quality assurance (QA) for a typical LDR BT treatment plan 1 month following implantation. Imaging the prostate (*red*) and rectum (*blue*) interface can be challenging on CT-guided post-implant QA. Isodose lines are shown as a percentage of the prescription dose (145 Gy). Sources are identified as *green circles* with *upward-directed* and *downward-directed triangles* representing out-of-plan sources. Treatment planning system is VariSeed 8.0 (Varian Inc. Palo Alto, CA, USA)

Typically, post-implant dosimetry is performed 1 month after an LDR BT implant using CT guidance (Fig. 7); the position of the implanted sources are identified, along with normal tissues and target volumes, and the isodose distribution is recomputed to evaluate the quality of the delivered treatment (Potters et al. 2001). Post-operative dosimetry metrics have been poorly correlated with intra-operative dosimetry; a study by Acher et al. (2010) showed that this was largely due to the subjective nature of the contoured prostate volume on CT (Merrick et al. 1999; Acher et al. 2010). MRI-based post-implant dosimetry has been proposed to offer improved discrimination between the prostate and OARs; however, signal voids around the metallic casings of brachytherapy sources pose a challenge for accurate source localization. Thomas et al. (2009) demonstrated that signal voids surrounding brachytherapy sources exist for increasing field strengths (3–4.5 mm separation for 1.5 T, and 4.5–6 mm for 3 T), which affect the accuracy with which the sources are localized; a proton density-weighted FSE sequence was used to limit this effect with good results (Thomas et al. 2009). Various studies demonstrated techniques to minimize these artifacts; Kuo et al. (2010) examined an in-phantom method using inversion recovery with ON-resonant suppression (IRON) to generate positive contrast in areas of high magnetic susceptibility artifact (Kuo et al. 2010). Similarly, positive contrast agents such as cobalt dichloride-N-acetyl-cysteine (C4) have been used as encapsulated markers and show promise at a number of different parameters and field strengths, without altering the dosimetry of the delivered therapy, and showing minimal patient toxicities (Lim et al. 2014; Frank et al. 2008).

## The future of targeted prostate brachytherapy

The advent of targeted imaging and treatment techniques has spawned interest in novel brachytherapy planning methodologies. The use of mpMRI for improved prostate and DIL localization may shift the focus of treatment to preferentially target focal lesions with higher radiation doses, while reducing doses to surrounding normal tissues. In addition to the improved localization offered by mpMRI, novel radiosensitizing nanoparticles have recently gained popularity as a method to selectively boost the dose of radiation to focal lesions beyond conventional means while maintaining normal tissue toxicities at current levels.

### Focal and salvage therapy using MRI guidance

Traditionally, the use of CT and TRUS guidance for prostate BT planning was limited to treating the entire gland, largely due to the inability to resolve the multifocal nature of intraprostatic disease with confidence. The significant improvement in the ability to differentiate individual focal lesions using mpMRI enables targeted dose escalation, while decreasing the whole-gland dose, thereby significantly reducing the dose to the OARs (Muller et al. 2014; Crehange et al. 2014).

Studies evaluating focal boosting to both single and multifocal DILs using both LDR and HDR have emerged recently. Ahmed et al. (2012) showed excellent 12 month outcomes and urinary toxicity following LDR BT focal boost (Ahmed et al. 2012). Banerjee et al. (2015) and Gomez-Iturriaga et al. (2016) demonstrated similar results with HDR BT showing significant escalation of DIL doses without an expected increase in urinary toxicities (Gomez-Iturriaga et al. 2016; Banerjee et al. 2015). This approach has also shown promise in focal salvage therapy, using both LDR and HDR, in reduction of urinary toxicities while maintaining excellent outcomes comparable to whole-gland salvage treatment (Hsu et al. 2013; Duijzentkunst et al. 2016).

These promising hypothesis-generating studies require robust long-term control and toxicity data, but they do indicate that focal boosting (and focal salvage therapy) using mpMRI-guided HDR and LDR is a feasible method for dose escalation while minimizing OAR toxicities.

There exist ample opportunities for multimodality fusion of mpMRI as well as intra-operative MRI for focal dose escalation. Significant technological hurdles to the implementation of intra-operative MRI-guided BT make it more likely that multimodality fusion will be readily adopted as a means to deliver focal or whole-gland BT. For centers that have the ability to implement intra-operative guidance for BT, it is likely that closed-bore, high-strength MRIs will see increased uptake due to their wider availability and ability to deliver high-quality anatomical and functional imaging.

### Radiosensitization using gold nanoparticles

Another promising new technique for improving local dose escalation, potentially acting synergistically with the improved image guidance afforded by mpMRI, is the use of gold nanoparticles (GNP) for selective radiosensitization of tumors (Jain et al. 2012; Babaei and Ganjalikhani 2014). The main mechanism of action of these nanoparticles is the production of photo- and Auger electrons by the photoelectric effect after bombardment with photons (Spiers 1949; Castillo et al. 1988). The short range of these electrons requires that cytosolic uptake of GNPs occurs to create DNA single- and double-strand breaks (Zheng et al. 2008). The selective dose enhancement factor (DEF)—the increased effect of local radiation dose deposition due to the GNPs—has been observed with high GNP concentrations in conjunction with keV photon energies as outlined by Zhang et al. 2008, Rahman et al. (2009) and Roeske et al. (2007). The introduction of GNP-mediated radiosensitization both for LDR and HDR brachytherapy, which emit gamma photon energies in the ideal range for maximizing DEF, holds significant promise.

Due to the long half-life of most LDR brachytherapy sources, GNPs introduced into cancerous cells must remain within the cell cytosol for extended periods of time to sufficiently provide a dose-enhancement effect. Shorter periods of GNP uptake by prostate cancer cells may be suitable for HDR delivery, due to the rapid radiation delivery (in the order of 10–30 min), and it therefore may serve as an easier pathway to GNP integration within the BT framework. Further development and customization of GNPs to specifically tailor them for use in prostate BT should focus on the route of administration, pharmacokinetics, and cellular uptake.

The geometric and functional parameters of GNPs play an important role in their uptake as outlined in Albanese et al. (2012), Perrault et al. (2009) and Favi et al. (2015). Naïve GNPs without additional ligands preferentially accumulate at sites of porous and leaky tumor vasculature (Jain et al. 2012). The addition of ligands such as polyethylene glycol (PEG) allow improved non-specific uptake by receptor-mediated endocytosis (RME) within tumors, as well as improved transit times in systemic circulation (Zhang et al. 2008; Kumar et al. 2013; Lechtman et al. 2013; Chithrani et al. 2006). Further functionalization by addition of tumor-specific ligands may enable GNPs to further target prostate cancer with increased specificity. Of importance in uptake and clearance is GNP size: smaller GNPs tend to have rapid circulations when administered intravenously and are quickly cleared by the renal system but more rapidly permeate tumor vasculature, while excessively large particles may have significantly increased uptake by the Reticuloendothelial System (RES), a process which decreases selective DEF (Arnida and Ghandehari 2010; Maeda et al. 2001).

In contrast to systemic administration, the intra-operative nature of BT procedures may enable interstitial injection of GNPs directly within focal lesions outlined on mpMRI. In this proposed workflow, the pre-treatment mpMRI-TRUS—or intra-operative MRI—could be used to localize focal lesions and the high spatial resolution of MRI could be used to guide deposition of high concentrations of GNPs directly within these cancerous foci during the BT procedure. This approach may significantly reduce the need for larger GNP sizes with their increased residence time, thereby taking advantage of the benefits of small GNP sizes. Evidence suggests that a medium range (6–50 nm) PEGylated, spherical GNP, administered interstitially, could allow sufficient tumor uptake and retention over the course of LDR BT to offer a significant dose-enhancement effect (Chithrani et al. 2006). For HDR BT, a smaller size (<6 nm) PEGylated, spherical GNP may be more suitable, allowing for a residence time and dispersion that correlated with the procedure duration, thereby producing the required dose enhancement with fewer potential side effects. GNPs could be administered during the procedure and rapidly cleared from circulation afterward. Commercially available GNPs specifically optimized for radiation therapy are also being developed to have longer circulation, small sizes (2–3 nm), higher tumor uptake, and improved clearance (Kumar et al. 2013). It remains to be seen if the pharmacokinetics of these commercially available GNPs is similar when administered locally within the prostate gland.

Conjugation of gadolinium with GNPs (Gd-GNP) could also allow for visualization on intra-operative MRI and offer a means of calculating the biological effective dose from the additive effects of GNPs during HDR BT (Harisinghani et al. 2003; Debouttiere et al. 2006; Le Duc et al. 2014). Vartholomeos et al. 2011 also examined the use of MRI-compatible nanoparticles to act as drug-delivery nanorobots under MRI-guided steering; similar methodologies may be applied to both track and steer injected GNPs to tumor vasculature within the prostate gland during BT (Vartholomeos et al. 2011).

There remain significant challenges with local deposition of high GNP concentrations within the prostate gland largely relating to their diffusion and uptake within the tumor vasculature. The assumption of homogenous GNP distribution with local administration may not be accurate, especially with local administration; problems with inconsistent tumor vasculature (particularly in hypoxic tumor regions) may cause poor uptake or heterogeneous GNP distribution even within small focal lesions. It is prudent to assume that focal lesions neighboring normal tissues, such as the urethra, may be at risk of excessive local hot spots with uneven GNP distribution and therefore further exploration is needed. Additionally, although systemic toxicities of GNP administration have been noted as minimal in some studies (Alkilany and Murphy 2010; Fratoddi et al. 2014), the examination of toxicities from localized injection of high GNP concentrations within the prostate requires further study if they are to be used in BT.

## Conclusions

Although long-term biochemical control and toxicity results of mpMRI-guided BT are forthcoming, it is expected that this method will continue to drive high-precision dose escalation for localized prostate cancer in the near future. The ability to deliver large localized doses to focal lesions within the prostate gland has profound implications for BT as first-line therapy as well as salvage therapy. Inclusion of GNPs to improve the radiosensitivity of prostate cancer is expected to offer additional normal tissue sparing effects and is a promising area of development. Future in-human clinical trials of radiotherapy-specific GNPs may shed some light on the impact these particles will have on prostate cancer.

## Abbreviations

ABS: American brachytherapy society
ADC: apparent diffusion coefficient
ADT: androgen deprivation therapy
BPH: benign prostatic hyperplasia
BT: brachytherapy
C4: cobalt dichloride-N-acetyl-cysteine
CT: computed tomography
DCEI: dynamic contrast-enhanced imaging
DEF: dose enhancement factor
DIL: dominant intraprostatic lesion
DWI: diffusion-weighted imaging
EBRT: external beam radiation therapy
GBCA: gadolinium-based contrast agent
GEC-ESTRO: Groupe Européen de Curithérapie/European society for radiotherapy and oncology
GNP: gold nanoparticle
Gy: gray
HDR: high-dose-rate brachytherapy
IRON: inversion recovery with ON-resonant suppression
LDR: low-dose-rate brachytherapy
mpMRI: multiparametric magnetic resonance imaging
MRSI: magnetic resonance spectroscopic imaging
OAR: organs at risk
PEG: polyethylene glycol
PI-RADS: prostate imaging reporting and data system
PSA: prostate-specific antigen
RES: reticuloendothelial system
RP: radical prostatectomy
T: tesla
T2W/FSE: T2-weighted MRI/fast-spin echo
TE: echo time
TR: repetition time
TRUS: TransRectal ultrasound
